# Risperidone stimulates food intake and induces body weight gain via the hypothalamic arcuate nucleus 5‐HT2c receptor—NPY pathway

**DOI:** 10.1111/cns.13281

**Published:** 2019-12-27

**Authors:** Xiao‐Qin Wan, Fan Zeng, Xu‐Feng Huang, He‐Qin Yang, Lan Wang, Yan‐Chuan Shi, Zhi‐Hui Zhang, Shu Lin

**Affiliations:** ^1^ Department of Cardiology Southwest Hospital Third Military Medical University (Currently Army Medical University) Chongqing China; ^2^ Illawarra Health and Medical Research Institute and School of Medicine University of Wollongong Wollongong NSW Australia; ^3^ Diabetes and Metabolism Division Garvan Institute of Medical Research Darlinghurst, Sydney NSW Australia; ^4^ Faculty of Medicine St Vincent's Clinical School UNSW Sydney Sydney NSW Australia

**Keywords:** 5‐HT2c receptor, hypothalamic arcuate nucleus (Arc), NPY, obesity, risperidone

## Abstract

**Aims:**

Many patients taking risperidone for the treatment of psychiatric disorders experience substantial body weight gain. Researchers have speculated that risperidone induces obesity by modulating central signals; however, the precise central mechanisms involved remain to be fully elucidated.

**Methods:**

Twenty‐four C57BL/6J mice were divided into four groups: a control group; a risperidone‐treated group; a lorcaserin‐treated group; and a combined risperidone + lorcaserin‐treated group. The mice were received the corresponding treatments for 4 weeks, and their brains were collected for in situ hybridization analysis. A subset of C57BL/6J mice was administrated with risperidone or placebo, and brains were collected 60 minutes post‐treatment for determination of c‐fos activity. In addition, brains of NPY‐GFP mice treated with or without risperidone were collected to perform colocalization of NPY and c‐fos, as well as NPY and 5‐HT2c receptor using immunohistochemistry.

**Results:**

There was significantly elevated c‐fos expression in the hypothalamic arcuate nucleus (Arc) of risperidone‐treated mice. More than 68% c‐fos‐positive neurons were NPY‐expressing neurons. Furthermore, in situ hybridization revealed that Arc NPY mRNA expression was significantly increased in the risperidone‐treated group compared with control group. Moreover, we identified that 95% 5‐HT2c receptors were colocalized with NPY positive neurons, and increased Arc NPY mRNA expression induced by risperidone was markedly reduced by cotreatment with lorcaserin, a specific 5‐HT2c receptor agonist.

**Conclusion:**

Our findings provide critical insight into the mechanisms underlying antipsychotic‐induced obesity, which may assist the development of therapeutic strategies to address metabolic side effects of risperidone.

## 1 INTRODUCTION

Second‐generation antipsychotics (SGAs) are used to effectively treat a variety of psychiatric disorders, including schizophrenia, bipolar disorder, autism spectrum disorder (ASD), and Alzheimer's disease.^1^ Most SGAs are associated with a wide range of potential adverse effects such as obesity, obesity‐associated dyslipidemia, and type 2 diabetes.^2^ In addition, life expectancy is reduced in patients with schizophrenia who have obesity‐related metabolic syndrome.^3^

Risperidone, a benzisoxazole derivative, is one of the SGAs with selective antagonistic properties at 5‐HT2c receptors.^4^ Over the past two decades, risperidone has been used to effectively treat a broad spectrum of psychiatric disorders.^5^ Indeed, risperidone is the most commonly prescribed antipsychotic medication among children and adolescents with bipolar disorder, schizophrenia, and attention‐deficit/hyperactivity disorder (ADHD).^6^, ^7^

Compared to first‐generation antipsychotics, second‐generation antipsychotics cause fewer extra‐pyramidal problems, but they present new challenges because they often lead to metabolic disorders. For example, risperidone increases food intake and induces weight gain, glucose intolerance, hypertriglyceridemia, and hyperprolactinemia.^8^, ^9^, ^10^ Previous research has suggested that risperidone induces weight gain by upregulating the expression of hypothalamic histaminergic H1 receptors and neuropeptide Y (NPY).^11^ Central NPY signaling is known to play a critical role in the regulation of food intake and energy balance,^12^ suggesting that preventing or reversing the increase in NPY expression may help reduce risperidone‐induced weight gain. Several main nuclei in the hypothalamus are involved in the regulation of body weight and food intake, including the hypothalamic arcuate nuclei (Arc), dorsomedial hypothalamic nuclei (DMH), and lateral hypothalamic area (LHA).^12^ In particular, NPY neurons in the Arc play a critical role in the control of energy homeostasis. The activation of NPY neurons in the Arc increases appetite.^13^, ^14^ These neurons project to other nearby hypothalamic areas to coordinate appetite control. For instance, Arc NPY neurons mediate the effect of LHA neurons on feeding behavior.^15^ Additional research has indicated that NPY neurons, which are abundantly expressed in the DMH following exposure to a high‐fat diet, exert their effects independently of sympathetic activity.^16^

In contrast to their non‐obesogenic counterparts, obesogenic SGAs are commonly associated with potent 5‐HT2c receptor antagonism.^17^ A previous study reported that 5‐HT2c receptors can inhibit the expression of orexigenic growth hormone secretagogue receptor 1a (GHSR1a) in the hypothalamus.^18^ GHSR1a stimulates food intake and fat deposition primarily via intracellular signaling pathways that increase orexigenic NPY and agouti‐related peptide (AgRP) expression while suppressing anorexigenic pro‐opiomelanocortin (POMC) signaling in the hypothalamus.^19^, ^20^, ^21^ GHSR1a antagonism reduces hypothalamic NPY expression, food intake, and body weight in rodents.^22^, ^23^ Thus, restoring the activity of 5‐HT2c receptors may prevent increases in NPY expression. Lorcaserin, an FDA‐approved anti‐obesity drug, is a selective 5‐HT2c receptor agonist. Therefore, we speculated that cotreatment with lorcaserin would prevent or reverse risperidone‐induced weight gain.

Based on these findings, we hypothesized that risperidone acts on the NPY system via 5‐HT2c receptors in the Arc to increase food intake and body weight. In the present study, we investigated the anatomic localization of 5‐HT2c receptors on NPY neurons in the Arc and the role of 5‐HT2c receptors in risperidone‐induced metabolic impairments in mice. We also aimed to determine whether combined treatment with a 5‐HT2c receptor agonist would restore 5‐HT2c receptor inhibitory control over the obesogenic GHSR1a, thereby averting the onset of hyperplasia and weight gain due to risperidone treatment.

## 2 MATERIALS AND METHODS

### 2.1 Animals

At 8 weeks of age, 24 female mice (C57BL/6J) were divided into 4 groups: a control group (n = 6), a risperidone only group (n = 6), a lorcaserin only group (n = 6), and a combined risperidone + lorcaserin group (n = 6). Food intake and body weight were measured weekly during the 4‐week experimental period, and the mouse brains were collected for in situ hybridization. Another 10 C57BL/6J mice were divided into risperidone‐treated mice (n = 5) and control mice (n = 5) for the detection of c‐Fos activity. In addition, 12 transgenic mice expressing GFP at NPY neurons (NPY‐GFP) were purchased from Jackson Laboratory were collected brains for double labeling immunohistochemistry (NPY/c‐Fos and NPY/5‐HT2c receptors).

### 2.2 Ethics and animal care

All experimental animal protocols were approved by the Third Military Medical University Animal Care Committee, in accordance with the National Institutes of Health Guide for the Care and Use of Laboratory Animals (NIH publication number: 8023). The 34 C57BL/6J mice and 12 NPY‐GFP mice were housed in temperature‐controlled (23 ± 2°C) and light‐controlled (12:12‐hours light‐dark cycle, lights on at 07:00 hours) animal quarters. Mice were provided ad libitum access to water and a standard chow diet.

### 2.3 Determining changes in food intake and body weight in response to risperidone treatment

At 8 weeks of age, 24 C57BL/6J mice were divided into four treatment groups. After acclimatization to the experimental conditions, treatments were administered at the same time every day for 4 weeks. One group of mice was intraperitoneally injected with risperidone (2 mg/kg), while the control group was treated with saline. One group of mice was intraperitoneally injected with risperidone + lorcaserin (10 mg/kg), and one group of mice was intraperitoneally injected with lorcaserin only. Drug dosages were based on those used in previous rodent studies.^24^, ^25^, ^26^ Body weight and food intake were measured weekly during the treatment period. Food intake was determined by calculating the difference between the given and remaining amounts of food after 24 hours. After the last experiments, brain tissue was collected for in situ hybridization and stored at −80°C until the assay.

### 2.4 Immunoreactivity for c‐Fos in the Arc, DMH, and LHA

We examined c‐Fos immunoreactivity in the brains of C57BL/6J mice intraperitoneally injected with risperidone (2 mg/kg) (n = 5) or saline (n = 5) 60 minutes after treatment. To detect c‐Fos expression in the hypothalamus, mice were anesthetized and perfused with saline followed by 4% paraformaldehyde via a cannula inserted into the left ventricle. After perfusion, the brains were immediately removed, kept in 4% paraformaldehyde, immersed in 30% sucrose, and stored at −80°C until use. Coronal brain sections (thickness: 30 µm) were obtained using a microtome (Menzel‐Glaser), following which c‐Fos expression was detected as previously described.^15^ The primary antibody (Cell Signaling Technology) was diluted at 1:400 in antibody buffer diluent. The brain sections were then incubated with secondary antibody against c‐Fos (Alexa Fluor^®^ 594 goat anti‐rabbit IgG, A11037, Life Technologies) for 3 hours. A Nikon orthotopic microscope was used to visualize red c‐Fos staining. Data were expressed as the average number of stained nuclei within the brain nuclei of interest and quantified using ImageJ software (NIH). To ensure consistency in the analysis of cell counts between the groups, sections through the Arc, DMH, and LHA of risperidone‐treated and control animals were examined as previously described.^27^

### 2.5 Double labeling of NPY neurons and c‐Fos immunoreactivity in the Arc of NPY‐GFP mice

Eight NPY‐GFP transgenic mice aged 10 weeks were purchased from Jackson Laboratory. The NPY‐GFP mice were treated with risperidone (n = 4) (2 mg/kg) or saline (n = 4) as previously mentioned. Sixty minutes later, mice were sacrificed, and their brains were immediately removed and placed on dry ice. The brain samples were then incubated with the primary antibody (rabbit‐anti‐mouse c‐Fos; 1:400) (Cell Signaling Technology), followed by a secondary antibody against c‐Fos to visualize red fluorescent staining (Alexa Fluor^®^ 594 goat anti‐rabbit IgG, A11037, Life Technologies), and a Nikon orthotopic microscope was used to visualize green fluorescent‐stained NPY neurons overlapping red c‐Fos‐stained neurons.

### 2.6 Double labeling of NPY neurons and 5‐HT2c receptor immunoreactivity in the Arc of NPY‐GFP mice

The remaining 4 NPY‐GFP mice were used to detect double labeling of NPY neurons and 5‐HT2c receptor immunoreactivity in the Arc. NPY‐GFP mice were sacrificed, following which their brains were removed and placed on dry ice. The brains were then incubated with the primary antibody (rabbit‐anti‐mouse 5‐HT2c receptor; 1:300) (Cell Signaling Technology), followed by a secondary antibody against 5‐HT2c receptor to visualize red fluorescent staining (Alexa Fluor^®^ 594 goat anti‐rabbit IgG, A11037, Life Technologies). A Nikon orthotopic microscope was used to visualize green fluorescent‐stained NPY neurons overlapping red 5‐HT2c receptor‐stained neurons.

### 2.7 In situ hybridization (ISH) using RNAscope technology

Brain samples collected from the four groups were used for ISH. Fresh brain tissue was cryosectioned at 20 µm and stored at −80°C. Transcript detection was performed using the commercially available RNAscope brown reagent kit (Advanced Cell Diagnostics). ISH was performed in accordance with the manufacturer's instructions for fixed‐frozen tissue. Detection experiments were performed in a hybridization oven (HybEZ™, ACD) with RNAscope Probe‐mm‐NPY (ACD 313321). Ppib probe (ACD 313911), a mouse housekeeping gene, was used as a positive control, while a bacterial dapB probe (ACD 310043) was used as a negative control. Each set of probes contained a tag that enabled visualization of the target transcript in brown. To compare differences in expression among the 4 groups, we quantified the integral optical density (IOD) of positive NPY staining using ImageJ, normalized by the stained area. The mean intensities from three random areas of the same size in the target areas were measured for each probe.

### 2.8 Statistical analyses

To determine the differences in calories intake, body weight and Arc NPY mRNA expression among four groups at the end of experiment, ordinary one‐way analysis of variance (ANOVA) with Tukey's post hoc test was used. To determine the difference between control and risperidone treatment group at various brain regions (Arc, DMH, and LHA), unpaired *t* test with two‐tailed *P* value was used (GraphPad Prism 5, version 5.0a; GraphPad Software). For all statistical analyses, the values were expressed as means ± SEM *P* values < .05 were considered statistically significant.

## 3 RESULTS

### 3.1 Risperidone stimulates food intake and increases body weight in mice

**Figure 1:**
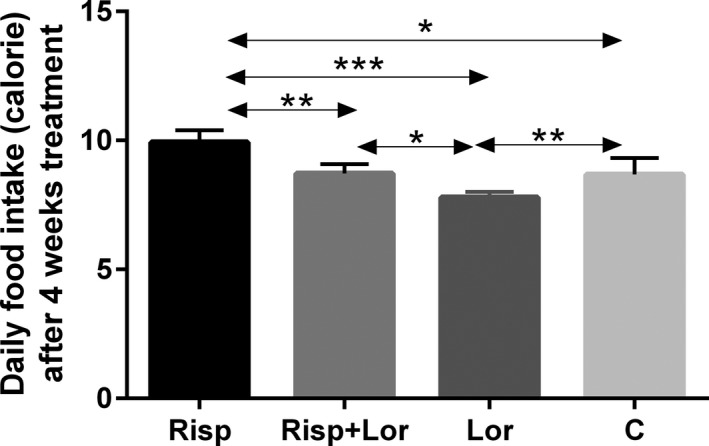
Daily food (calorie) after 4‐wk treatment. Results are shown as mean ± SEM of 4‐6 mice per group. *P < .05; **P < .01; ***P < .001. Risp: risperidone‐treated group; Lor: lorcaserin‐treated group; Risp + Lor: risperidone + lorcaserin‐treated group; C: control group

**Figure 2:**
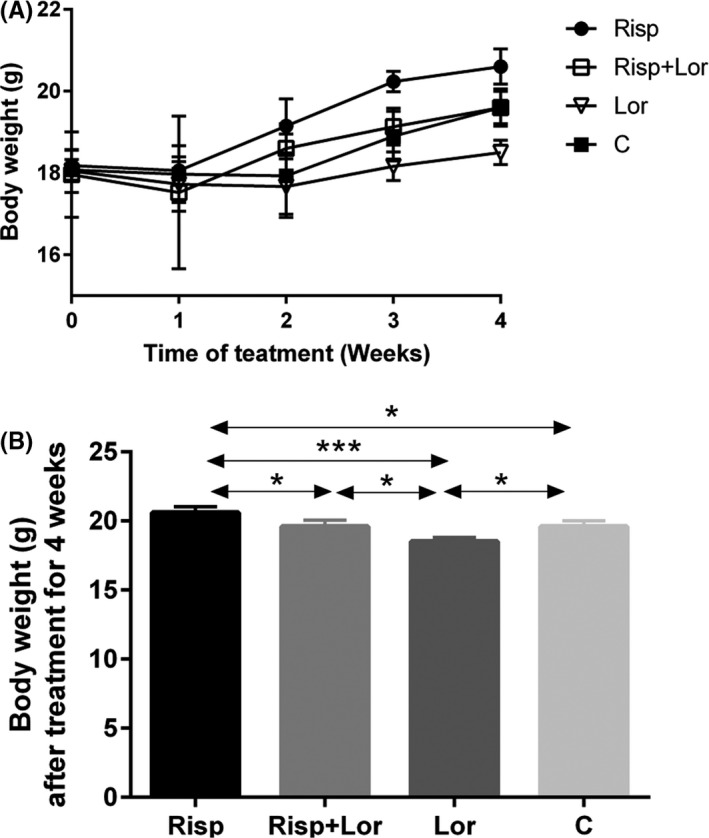
Body weight after treatment. A, The body weight measured weekly during treatment. B, Body weight after treatment for 4 wks. Results are shown as mean ± SEM of 4‐6 mice per group,*P < .05; **P < .01; ***P < .001. Risp: risperidone‐treated group; Lor: lorcaserin‐treated group; Risp + Lor: risperidone + lorcaserin‐treated group; C: control group

During the 4‐week experiment, food intake and body weight increased over time in all groups. At the end of experiment, significant differences in calorie intake were observed between the risperidone‐treated groups and control groups (risperidone vs control, 10 ± 0.209 vs 8.6 ± 0.363 kcal, *F* value (3,12) = 19.01, *P* = .018) (Figure 1, Table 1). Also, there was significant difference in body weight between these two groups (risperidone vs control, 20.6 ± 0.216 vs 19.6 ± 0.231 g; *F* value (3,9) = 15.39, *P* = .026) (Figure 2, Table 1).

**Table 1:** The calorie intake, body weight, and the Arc mRNA expressions after 4‐wk treatment and the c‐fos expression in the brain area of Arc, DMH, and LHA after 60‐min treatment

	Control	Risperidone	Risperidone + Lorcaserin	Lorcaserin
Body weight (g)	19.6 ± 0.231*	20.6 ± 0.216	19.6 ± 0.265**	18.5 ± 0.173***
Calorie intake (kcal)	8.6 ± 0.363*	10 ± 0.209	8.7 ± 0.183**	7.7 ± 0.107***
c‐Fos in the Arc	43.6 ± 3.572#	73.6 ± 4.02	/	/
c‐Fos in the DMH	42 ± 2.775#	115 ± 6.066	/	/
c‐Fos in the LHA	35 ± 4.946#	70.8 ± 5.389	/	/
mRNA in the Arc	22.4 ± 1.749*	32.2 ± 2.634	22.6 ± 1.536**	13.4 ± 0.927***

Note

Data are means ± SEM, n = 4‐6 for each group. The differences were determined by either one‐way ANOVA with Tukey post hoc test for body weight and calorie intake or unpair *t* test for c‐fos analysis.

* *P* < .05 vs risperidone or lorcaserin.

** *P* < .05 vs risperidone or lorcaserin.

*** *P* < .05 vs risperidone.

^#^ *P* < .05 vs risperidone.

### 3.2 Risperidone activates c‐Fos expression in the hypothalamic Arc, DMH, and LHA

**Figure 3:**
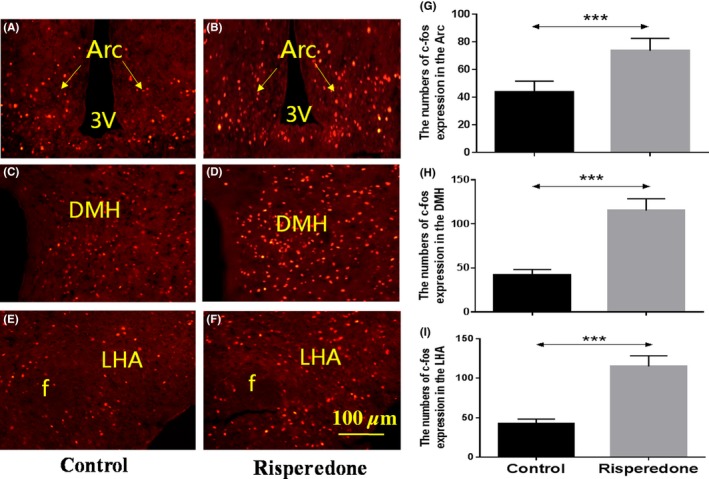
The expression of c‐Fos was increased in the Arc, DMH, and LHA after risperidone treatment for 60 min. The expression of c‐fos of C57BL/6J mice treated with saline in the Arc (A), DMH (C) and LHA (E). The expression of c‐Fos of C57BL/6J mice treated with risperidone in the Arc (B), DMH (D), and LHA (F). G, H, I results are shown as means ± SEM of 5 mice per group. ***P < .001, control group vs risperidone‐treated group. Arc: arcuate hypothalamic nucleus; DMH: dorsomedial hypothalamic nuclei, LHA: lateral hypothalamic area, 3V: the third cerebral ventricle, f: fornix, Scale bar = 100 µm

We examined c‐Fos expression to determine which brain regions were activated in response to risperidone treatment. The numbers of c‐Fos‐labeled neurons in risperidone‐treated mice vs control mice were 73.6 ± 4.02 vs 43.6 ± 3.572 in the Arc (Figure 3A and B, *P* = .001); 115 ± 6.066 vs 42 ± 2.775 in the DMH (Figure 3C and D, *P* < .001); and 70.8 ± 5.389 vs 35 ± 4.946 in the LHA (Figure 3E and F, *P* = .001).

### 3.3 NPY neurons colocalize with c‐Fos‐positive neurons in the Arc following risperidone treatment

**Figure 4:**
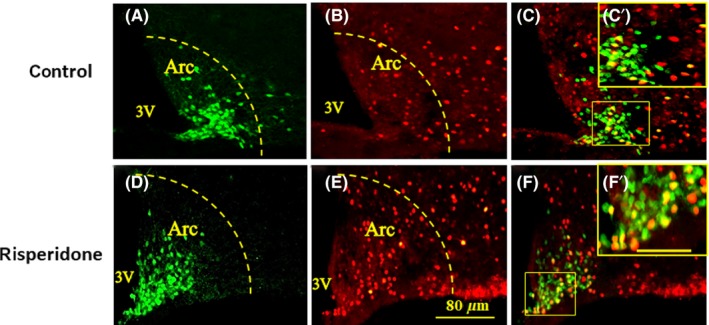
Fluorescent immunohistochemical detection of c‐Fos and GFP‐NPY neurons coexpressed in the hypothalamic arcuate nucleus at 60 min after saline (A‐C) or risperidone (D‐F) treatment. A and D, Green staining shows NPY neurons expressing GFP; (B, E): red staining shows c‐Fos‐positive neurons. C and F, Yellow staining indicating that the neurons express both c‐fos and NPY; C'&F' is higher magnification of the boxed area in C&F. Scale bars in A‐F is 80 μm and in C'&F' is 25 μm

To confirm whether c‐Fos‐positive neurons exhibit a relationship with NPY neurons, we examined c‐Fos immunoreactivity in NPY‐GFP mice. Sixty minutes after risperidone or saline treatment, we observed that 68% of c‐Fos‐positive neurons overlapped with NPY‐GFP neurons in the Arc in the risperidone group, while the overlapping ratio in the control group was only 21% (Figure 4).

### 3.4 Risperidone increases the expression of NPY mRNA in the Arc

**Figure 5:**
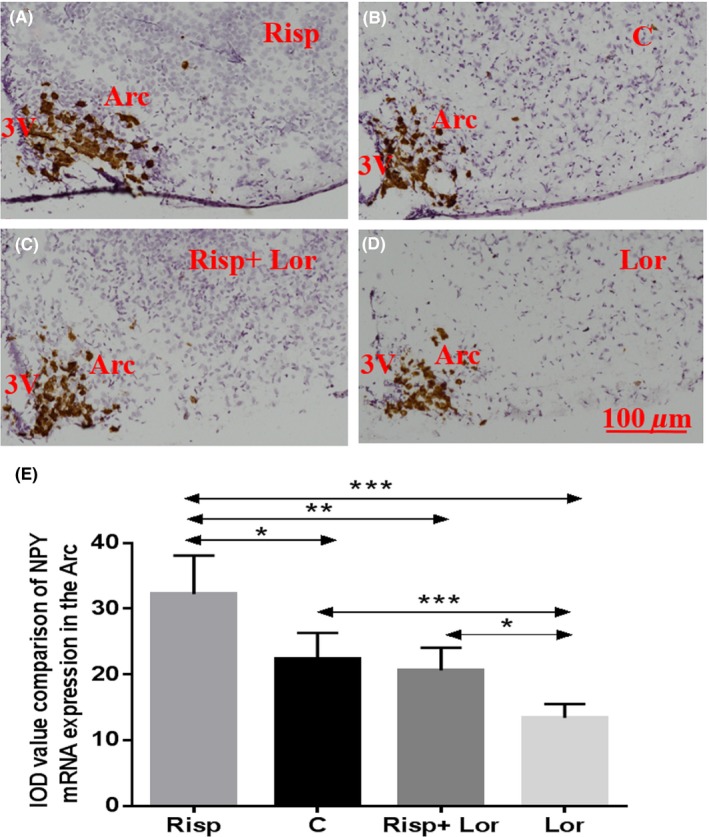
Upregulated NPY mRNA expression in the Arc caused by risperidone treatment and downregulated NPY mRNA expression in the Arc after treated by lorcaserin; (A) NPY mRNA expression in the Arc of risperidone‐treated mouse; (B) NPY mRNA expression in the Arc of control mouse; (C) NPY mRNA expression in the Arc of risperidone + lorcaserin‐treated mouse; (D) NPY mRNA expression in the Arc of lorcaserin‐treated mouse; (E) data are means ± SEM of 6 mice per group. *P < .05, **P < .01, ***P < .001, control group vs risperidone‐treated group. 3V: the third ventricle; Arc: arcuate hypothalamic nucleus; Risp: risperidone‐treated group; Lor: lorcaserin‐treated group; Risp + Lor: risperidone + lorcaserin‐treated group; C: control group; Scale bar = 100 µm

Since Arc neurons are activated by risperidone, we examined NPY mRNA expression in Arc neurons via ISH. The NPY mRNA expression in the Arc was significantly higher in the risperidone‐treated group than in the control group (Figure 5).

### 3.5 5‐HT2c receptors colocalize with NPY neurons in the Arc of NPY‐GFP mice

**Figure 6:**
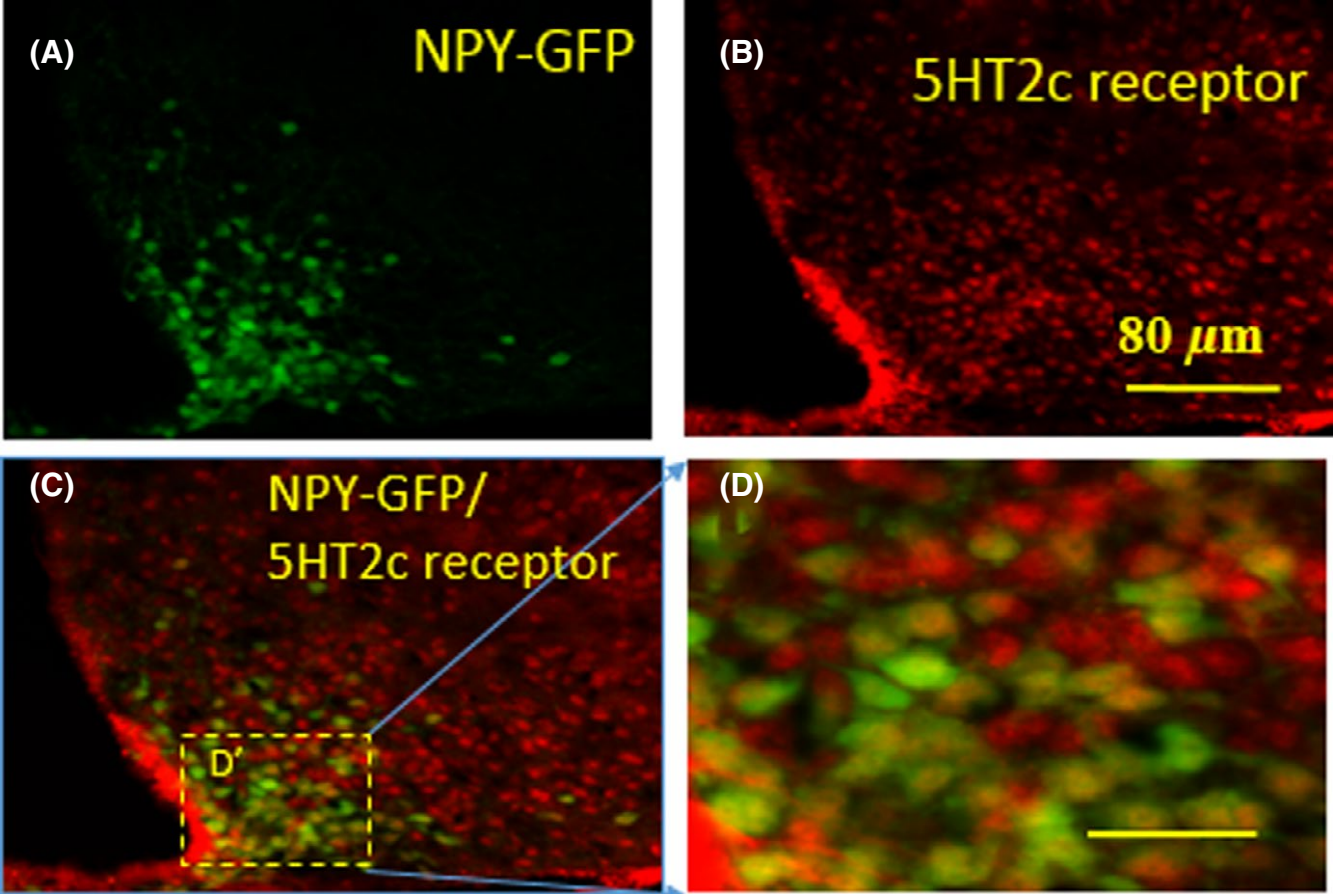
Fluorescent immunohistochemical detection of 5HT2c receptor and NPY neurons coexpressed in the Arc. A, Green staining shows NPY neurons expressing GFP; (B): red staining shows the 5HT2c receptors; (C): yellow staining indicating that the neurons express both 5HT2c receptors and NPY; (D) is higher magnification of the boxed area in D’. Scale bars in A, B, and C is 80 μm and in D is 25 μm

To determine whether 5‐HT2c receptors colocalize with NPY neurons in the Arc, we examined 5‐HT2c immunoreactivity in NPY‐GFP mice via double‐receptor labeling experiments. Our findings indicated that 95% of 5‐HT2c receptor overlapped with NPY‐GFP neurons in the Arc (Figure 6).

### 3.6 Lorcaserin counteracts risperidone‐induced increases in NPY mRNA expression in the Arc

Treatment with the selective 5‐HT2c receptor agonist lorcaserin led to weight loss (lorcaserin vs control, 18.5 ± 0.173 vs 19.6 ± 0.231 g, *F* value (3,9) = 15.39, *P* = .019) and alleviated weight gain caused by risperidone (lorcaserin + risperidone vs control, 19.6 ± 0.265 vs 19.6 ± 0.231 g, *F* value (3,9) = 15.39, *P* = .865) (Figure 2). Therefore, we examined levels of NPY mRNA expression in Arc neurons in the lorcaserin + risperidone group and the lorcaserin only group. The Arc NPY mRNA expression was lower in the lorcaserin only group than in the control group (lorcaserin vs control, 13.4 ± 0.927 vs 22.4 ± 1.749, *F* value (3,16) = 18.16, *P* = .014). However, no obvious differences in NPY mRNA expression were observed between the lorcaserin + risperidone group and the control group lorcaserin + risperidone vs control, 22.6 ± 1.536 vs 22.4 ± 1.749, *F* value (3,16) = 18.16, *P* = .9) (Figure 5, Table 1).

## 4 DISCUSSION

In the current study, we investigated the role of 5‐HT2c receptors in risperidone‐induced metabolic impairments, as well as the ability of a 5‐HT2c receptor agonist to attenuate these impairments. Our findings indicated that mice exhibited significant increases in body weight and daily food intake after 4 weeks of risperidone treatment. In the risperidone group, more than half of neurons expressing the c‐Fos gene colocalized with NPY, and almost all neurons expressing 5‐HT2c receptors colocalized with NPY. In addition, treatment with the 5‐HT2c receptor agonist lorcaserin significantly decreased the expression of NPY mRNA in the Arc. Such findings suggest that risperidone induces hyperphagia and obesity via the 5‐HT2c receptor‐NPY pathway in the Arc.

Risperidone stimulates food intake and increases body weight by affecting signals passing through the “hypothalamic feeding center”.^28^ Interactions between hypothalamic neural networks and peripheral positive energy balance rely on neuronal pathways that are involved in autonomic outflow from the brain. NPY in the Arc is a main central food stimulator responsible for restoring energy balance. Furthermore, NPY neurons from the Arc that project to the LHA play a major role in the regulation of food intake,^29^ while those that project to the DMH aid in reducing energy expenditure.^15^ Previous research has suggested that Arc NPY signaling is the most potent stimulator of food intake, exerting its effects by binding to Y1 receptors in the paraventricular nucleus (PVN).^14^ In the current study, risperidone treatment significantly increased c‐Fos expression in the Arc, indicating that risperidone initially activates neurons concentrated within this nucleus. In addition, we observed that 68% of c‐Fos‐positive neurons overlapped with NPY‐GFP neurons in the Arc in NPY‐GFP mice, suggesting that risperidone activates NPY neurons in the Arc. In order to determine whether Arc NPY neurons express 5‐HT2c receptors, we performed double‐labeling experiments to examine 5‐HT2c immunoreactivity in NPY‐GFP mice. To the best of our knowledge, the present study is the first to report that 95% of 5‐HT2c receptors overlapped with NPY‐GFP neurons in the Arc. Risperidone is a potent 5‐HT2c receptor antagonist, and previous studies have demonstrated that obesogenic antipsychotic drugs reduce 5‐HT2c receptor inhibition of GSHR1a.^17^ Therefore, our results indicate that risperidone may selectively stimulate NPY expression in the Arc by reducing 5‐HT2c receptor activity, thereby increasing food intake.

Research has revealed that NPY neurons in the Arc project to downstream LHA neurons via Y1 receptors, which play a crucial role in modulating feeding behavior.^30^ In the present study, abundant c‐Fos expression was observed in the LHA 60 minutes after risperidone treatment. Such increases were likely responsible for the observed increases in food intake among risperidone‐treated animals, as previous studies have indicated that orexin and melanin‐concentrating hormone (MCH) are also abundantly expressed in the LHA. Indeed, both orexin and MCH stimulate feeding in rats when injected intracerebroventricularly (ICV).^31^, ^32^

Weight gain is caused by either increased food intake, decreased energy expenditure, or both. Recent studies have begun to decipher the pathways responsible for energy homeostasis, and several groups are actively engaged in the development of pharmacological agents targeting these pathways. Such findings are in accordance with the observed increases in c‐Fos immunoreactivity in the DMH 60 minutes after risperidone treatment. Arc neurons send extensive projections to the DMH. These neuronal projections are important routes for leptin, insulin, and ghrelin in the hypothalamus.^33^ Researchers have speculated that Arc NPY neurons projecting to the DMH control energy balance by stimulating food intake and inhibiting heat generation, especially in the case of energy deficiency.^34^ It is well known that a set of neurons distributed in the DMH is responsible for these functions (eg, cocaine‐ and amphetamine‐regulated transcript (CART) neurons and cholinergic neurons). Further studies are required to identify the central mechanisms underlying changes in signaling between Arc NPY neurons and the DMH during risperidone treatment. Such studies may aid in the development of better antipsychotics with fewer side effects.

Notably, our findings indicated that treatment with lorcaserin attenuated risperidone‐induced increases in NPY mRNA expression in the mouse Arc. Lorcaserin, an FDA‐approved anti‐obesity drug, is a selective 5‐HT2c receptor agonist that also inhibits ghrelin signaling.^18^ A previous randomized, double‐blind, placebo‐controlled clinical trial involving 2,200 individuals with excessive weight or obesity indicated that lorcaserin treatment was associated with 5%‐10% weight loss over a follow‐up period of 1 year.^35^ Therefore, cotreatment with lorcaserin may restore 5‐HT2c receptor activity and prevent the initial disruption of GHSR1a‐induced appetite signaling caused by risperidone, which may in turn avert the onset of hyperphagia and weight gain. Huang et al reported that treatment with a GHSR1a antagonist inhibits GSHR1a signaling and prevents olanzapine‐induced hyperphagia in rats.^23^, ^36^ However, widespread GHSR1a blockade may not be ideal for combating SGA‐induced obesity given the beneficial effects of ghrelin on cognitive function.^37^ However, restoring normal hypothalamic GHSR1a activity by targeting the 5‐HT2c receptor‐GHSR1a dimer may directly address the mechanisms underlying olanzapine‐induced obesity. In addition, accumulating evidence has demonstrated that 5‐HT2c agonists exhibit procognitive effects,^38^, ^39^ suggesting that the use of 5‐HT2c receptor agonists can combat both obesity and psychiatric symptoms.

Our present study identifies a novel neuronal 5‐HT2c receptor‐NPY pathway via which antipsychotic medication risperidone increases energy intake and promotes body weight gain, and cotreatment with lorcaserin attenuates these side effects. This study highlights the importance of 5‐HT2c receptors in Arc NPY neurons in mediating risperidone's action on weight gain. It is worthwhile noting that an earlier study has shown that 5‐HT2c receptors in POMC neurons only is sufficient to mediate effects of serotoninergic compounds on food intake^40^; therefore, it is possible that the 5‐HT2c receptors in other neurons, for instance, Arc POMC neurons, may also play a role in risperidone‐induced body weight gain. Further studies are warranted to identify the involvement of 5‐HT2c receptors in other brain regions. Indeed, Pérez‐Maceira and colleagues demonstrate that 5‐HT2c receptor agonists MK212 does not alter hypothalamic AgRP mRNA expression in rainbow trout^41^; therefore, 5‐HT2c receptor in AGRP neurons may not play a main role in the risperidone‐induced weight gain. In addition, risperidone is antagonism of dopamine D2 receptors, which could lead to reduced voluntary activity and energy expenditure^42^, ^43^; therefore, the blockade of dopamine D2 receptors by risperidone may also contribute to risperidone‐induced weight gain. Similarly, there is evidence showing that risperidone significantly elevates mRNA expression of hypothalamic histamine H1 receptor (H1R), leading to hyperphagia and body weight gain in rats,^11^ which suggests an involvement of hypothalamic H1R in risperidone‐induced hyperphagia and obesity. Thus, it becomes evident that risperidone causes weight gain and obesity via complex mechanisms involving multiple neuronal pathways. Together, our present study unravels a key novel neural circuitry via which risperidone induces its metabolic side effects. Our findings will provide important evidence for the development of novel therapeutics to treat the SGAs‐associated metabolic disorders.

## CONFLICT OF INTEREST

The authors declare no conflict of interest.

## AUTHOR CONTRIBUTIONS

S L., Y‐C S., and Z‐H Z. conceived, designed and directed this research, and wrote and finalized the manuscript. X‐F H. provided critical discussions. X‐Q W. and F Z. performed experiments and drafted the manuscript. H‐Q Y. and L W. performed experiments. All authors read and approved the final manuscript. We would like to thank Editage [http://www.editage.cn] for English language editing.
